# P-1515. The Socioeconomic Value of Adult Respiratory Vaccination in the United States: A Benefit-Cost Analysis

**DOI:** 10.1093/ofid/ofaf695.1699

**Published:** 2026-01-11

**Authors:** Tianyan Hu, Matthew W Napier, Alon Yehoshua, Jeffrey T Vietri, Claud Theakston, Sulayman Z Chowdhury, Diana Mendes, Simon Brassel, Raymond Farkouh, Lotte Steuten

**Affiliations:** Pfizer Inc., New York, New York; Office of Health Economics, London, England, United Kingdom; Pfizer Inc., New York, New York; Pfizer, Inc., Collegeville, Pennsylvania; Office of Health Economics, London, England, United Kingdom; Office of Health Economics, London, England, United Kingdom; Pfizer Ltd., Tadworth, England, United Kingdom; Office of Health Economics, London, England, United Kingdom; Pfizer, Inc., Collegeville, Pennsylvania; Office of Health Economics, London, England, United Kingdom

## Abstract

**Background:**

In the United States, protection from respiratory diseases via vaccination is recommended against seasonal influenza (flu) and COVID-19 for adults aged 18+, Pneumococcal Disease (PD) for adults 50+ and at-risk adults aged 18-49, and Respiratory Syncytial Virus (RSV) for adults 75+ and at-risk adults aged 60-74.

Each of these respiratory vaccines can reduce mortality, morbidity, as well as burdens on the healthcare system, and evaluations have found them cost-effective from payer and societal perspectives.

However, there is currently a lack of evidence evaluating the combined socioeconomic value of adult respiratory vaccination.

**Methods:**

We quantified the socioeconomic value of vaccinating adults against PD, RSV, flu, and COVID-19 in the US, using an established Benefit-Cost Analysis (BCA) framework.

This included valuing i) mortality risk reduction using the Value of a Statistical Life approach, ii) morbidity risk reduction using the Cost-of-Illness approach, and iii) changes in time use using formal work productivity losses.

Four static, deterministic disease models were developed to estimate outcomes. We compared a vaccination strategy reflecting the current Advisory Committee on Immunization Practices’ vaccine recommendations to a comparator strategy of no vaccination.

We calculated Benefit-Cost Ratios (BCRs) and Net Benefits (NBs) by aggregating benefits and costs across all cohorts and programs at years 1, 5, 10 and over the lifetime.

**Results:**

At year 1, all programs combined avert over 12,000 deaths, free up more than 1.2 million hospital bed days and 3.6 million outpatient visits. This results in NBs of $132.5 billion, corresponding to a BCR of 5:1. NBs grow over time to $659.8 billion in year 5 (BCR 7:1), $1.3 trillion in year 10 (BCR 7:1) and to over $5.1 trillion (BCR 9:1) over all cohorts’ lifetimes.

**Conclusion:**

Adult respiratory vaccination programs generate a significant socioeconomic value with benefits greatly exceeding their costs. This study informs US decision-makers of the societal return on investment, and advocates for sustained adult vaccine recommendations to prevent respiratory diseases.

**Disclosures:**

Tianyan Hu, PhD, Pfizer: Salary|Pfizer: Stocks/Bonds (Public Company) Matthew W. Napier, MSc Health Economics and Health Policy, Amicus Therapeutics: Grant/Research Support|Astellas: Grant/Research Support|MSD: Grant/Research Support|Organon: Grant/Research Support|Pfizer: Grant/Research Support|Takeda: Grant/Research Support|ViiV: Grant/Research Support Alon Yehoshua, PharmD, MS, Pfizer Inc: Employee of Pfizer and may hold stock or stock options of Pfizer Jeffrey T. Vietri, PhD, Pfizer Inc: Employment|Pfizer Inc: Stocks/Bonds (Public Company) Claud Theakston, MSc, Grunenthal: Grant/Research Support|J&J: Grant/Research Support|MSD: Grant/Research Support|Novartis: Grant/Research Support|Pfizer: Grant/Research Support|Roche: Grant/Research Support|Sanofi: Grant/Research Support|Santen: Grant/Research Support Sulayman Z. Chowdhury, MSc, Big Health: Grant/Research Support|Grunenthal: Grant/Research Support|Novartis: Grant/Research Support|Pfizer: Grant/Research Support Diana Mendes, PhD, Pfizer: Salary|Pfizer: Stocks/Bonds (Private Company) Simon Brassel, MSc, Dipl.-Ing., GSK: Grant/Research Support|Johnson & Johnson: Grant/Research Support|Moderna: Grant/Research Support|Pfizer: Grant/Research Support|Sanofi: Grant/Research Support|ViiV: Grant/Research Support Raymond Farkouh, PhD, Pfizer: Stocks/Bonds (Public Company) Lotte Steuten, MSc, PhD, Amgen: Grant/Research Support|Astra Zeneca: Grant/Research Support|Biomerieux: Grant/Research Support|GSK: Grant/Research Support|Moderna: Grant/Research Support|MSD/Merck: Grant/Research Support|Pfizer: Grant/Research Support|ViiV: Grant/Research Support

